# Physical activity and activity space in patients with pulmonary fibrosis not prescribed supplemental oxygen

**DOI:** 10.1186/s12890-017-0495-2

**Published:** 2017-11-23

**Authors:** Elisabeth Dowling Root, Bridget Graney, Susan Baird, Tara Churney, Kailtin Fier, Majorie Korn, Mark McCormic, David Sprunger, Tomas Vierzba, Frederick S. Wamboldt, Jeffery J. Swigris

**Affiliations:** 1 Department of Geography and Division of Epidemiology, The Ohio State University, 1036 Derby Hall, 154 N. Oval Mall, Columbus, OH 43210 USA; 2 Division of Pulmonary Sciences and Critical Care Medicine, University of Colorado Anschutz Medical Campus, 1400 Jackson Street, Denver, CO 80206 USA; 3 Participation Program for Pulmonary Fibrosis (P3F), National Jewish Health, 1400 Jackson Street, Denver, CO 80206 USA; 4 Interstitial Lung Disease Program, National Jewish Health, 1400 Jackson Street, Denver, CO 80206 USA; 5 Division of Pulmonary, Critical Care and Sleep Medicine, Sleep & Behavioral Health Sciences Section, National Jewish Health, 1400 Jackson Street, Denver, CO 80206 USA

## Abstract

**Background:**

Patients with pulmonary fibrosis (PF) have impaired quality of life, and research suggests that dyspnea and physical activity are primary drivers. As PF progresses, some patients notice the disease “shrinks their worlds”. The objective of this study is to describe movement (both physical activity and activity space) in a cohort of patients with PF of various etiologies who have not been prescribed supplemental oxygen (O_2_).

**Methods:**

Subjects with PF not on supplemental O_2_ during the day were enrolled from across the U.S. from August 2013 to October 2015. At enrollment, each subject completed questionnaires and, for seven consecutive days, wore an accelerometer and GPS tracker.

**Results:**

One hundred ninety-four subjects had a confirmed diagnosis of PF and complete, analyzable GPS data. The cohort was predominantly male (56%), Caucasian (95%) and had idiopathic pulmonary fibrosis (30%) or connective tissue disease related-PF (31%). Subjects walked a median 7497 (interquartile range [IQR] 5766-9261) steps per day. Steps per day were correlated with symptoms and several quality of life domains. In a model controlling for age, body mass index, wrist- (vs. waist) worn accelerometer and percent predicted diffusing capacity (DLCO%), fatigue (beta coefficient = −51.5 ± 11.7, *p* < 0.0001) was an independent predictor of steps per day (model R^2^=0.34).

**Conclusions:**

Patients with PF, who have not been prescribed O_2_ for use during the day, have wide variability in their mobility. Day-to-day physical activity is related to several domains that impact quality of life, but GPS-derived activity space is not. Wearable data collection devices may be used to determine whether and how therapeutic interventions impact movement in PF patients.

**Trial registration:**

NCT01961362. Registered 9 October, 2013.

**Electronic supplementary material:**

The online version of this article (10.1186/s12890-017-0495-2) contains supplementary material, which is available to authorized users.

## Background

Pulmonary fibrosis (PF) is a lung condition characterized by diffuse, irreversible parenchymal scarring. PF may be associated with connective tissue diseases or occupational/environmental exposures. And for many patients, despite exhaustive evaluation, the underlying cause remains unknown; in such cases, patients are often diagnosed with an idiopathic interstitial pneumonia (IIP), of which the most common is idiopathic pulmonary fibrosis (IPF). PF usually presents with the insidious onset of shortness of breath; progression is common, and in all too many cases PF leads to early death. Patients with PF experience a number of intrusive symptoms; fatigue, a dry, hacking cough, along with ubiquitously-present, activity-limiting dyspnea all contribute to overall impaired quality of life (QOL) [1, 2]. As there are currently no therapies that reverse fibrosis, the primary goals of intervention are to slow progression, decrease symptoms and improve QOL.

Dyspnea is the strongest driver of QOL impairment in patients with PF [3]. Even in PF’s earlier stages, shortness of breath may force patients to slow down or, reluctantly, give up certain physical activities they enjoy. In the latter stages of PF, dyspnea can make even activities-of-daily-living feel like a “chore” [2, 4]. Many late-stage PF patients suffer from something we have labeled “the shrinking world syndrome”: the perception that they are literally and figuratively tethered to home and unable to move in the world as much as they desire [4]. We believe the “shrinking world” experience is quantifiable: one metric, “activity space”, is used by researchers in a variety of disciplines to define the local area within which people move or travel. Specifically, it represents the spatial movement component of an individual’s day-to-day lived experience and incorporates their living environment, individual capability for mobility, constraints, needs, preferences and resources available for movement. Although earlier in the course of PF – and particularly if supplemental oxygen (O_2_) is not needed – patients’ activity spaces may not have changed from prior to diagnosis (or even symptom onset), and their worlds may not have shrunken to any noticeable degree. However, this has never been studied.

A clearer understanding of PF patients’ physical activity level and activity space (i.e., movement), and precisely how they relate to other patient-centered outcomes, could improve communication between PF patients and their providers and help to identify targets for therapeutic intervention. In this study (conducted within our Patient Centered Outcomes Research Institute-funded research program, the Participation Program for Pulmonary Fibrosis), we aimed to quantify movement at baseline via metrics of physical activity and activity space, in a heterogeneous sample of PF patients who did not require O_2_ during the day and were enrolled in a study of O_2_ therapy in patients with PF. We hypothesized that physical activity would be at least moderately strongly associated with symptoms and QOL and that, because PF was relatively mild and/or subjects were early in the course of their disease as indicated by the absence of the need for O_2_ during the day, activity space would not be correlated with those outcomes.

## Methods

### Subjects

A full accounting of the methods for the study were published previously [5]. Briefly, between August 2013 and October 2015, patients with PF of any etiology, greater than 18 years of age, not using O_2_ during the day and able to speak and read English, were recruited either from the Interstitial Lung Disease Clinic at National Jewish Health (NJH) or through the P3F using an online recruitment strategy (http://www.pulmonaryfibrosis.org/life-with-pf/clinical-trials). We enrolled a total of 300 subjects. All subjects gave written, informed consent including consent to publish study results. The study was approved by the NJH Institutional Review Board (HS-2790), and the study is registered on ClinicalTrials.gov (NCT01961362).

### Data capture and outcome measures

At enrollment, participants were administered a series of questionnaires and sent an accelerometer and GPS logger to wear for a period of a week. Questionnaires, administered electronically via REDCap (https://www.project-redcap.org/), included the University of California San Diego Shortness of Breath Questionnaire (UCSD), Version 1 of the Medical Outcomes Study Short Form 36-Item Instrument (SF-36), the Fatigue Severity Scale (FSS), and the Leicester Cough Questionnaire (LCQ). The UCSD is a 24-item dyspnea questionnaire with scores ranging from 0 to 120 and higher scores indicating greater dyspnea [6]. The SF-36 is a generic health-related QOL (HRQL) questionnaire with eight domains and two component summaries (physical and mental). Each domain and component score was transformed to a scale in which respondents from the 1998 U.S. general population had a mean of 50 and a standard deviation of 10. Higher scores indicate greater HRQL [7]. The FSS is a 9-item questionnaire, scored from 9 to 63, with higher scores indicating more severe fatigue. The LCQ is a 19-item questionnaire that taps the physical, psychological and social aspects of cough. Domain scores range from 1 to 7 (total 3-21), with higher scores indicating better cough-related QOL [8].

We measured day-to-day physical activity with an accelerometer, the Actigraph GT3X + Tri-Axis Actigraphy Monitor (Actigraph LLC; Pensacola, FL) worn either on the wrist or waist using the provided manufacturer’s bands. We used the iGotU GT-600 GPS data-logger from MobileAction Technologies (http://global.mobileaction.com/support/support_igotU_Faq.jsp) to capture GPS data. The Actigraph and GPS were mailed to subjects who were asked to wear them for seven consecutive days (including at least one weekend day), then returned them in pre-addressed envelopes. During the 7-day study period, participants were also asked to record in an activity diary the start and end times of wearing the GPS and whether/when they left their home. The GPS was programmed to record position every 15 s and automatically stop logging points during periods of prolonged inactivity (e.g., 15 min or more) to conserve battery and memory. Participants were asked to charge the GPS unit at night while sleeping.

### Data processing and statistical analysis

**Fig. 1:**
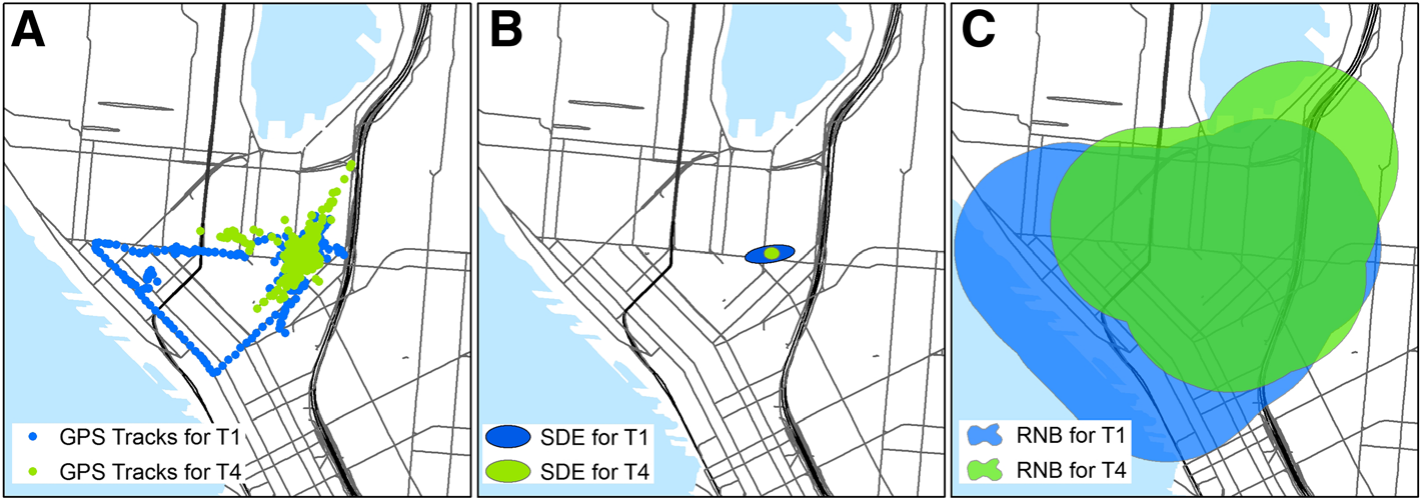
GPS data from one subject at two time points. Panel **a**. GPS tracks. Panel **b**. SDE for the same subject. Panel **c**. RNB for the Footnote: SDE = standard deviational ellipse; RNB = road network buffer. Data from two timepoints are given to show capabilities of data. Blue = data collected at baseline; green = data collected after supplemental oxygen was started. For both SDE and RNB, larger areas indicated greater activity space

GPS and accelerometer data were processed and linked to study participant data and questionnaires. We used generally accepted criteria for identifying participants with a valid GPS record: 1) had at least 4 days of recorded wear time and 2) the unit captured at least 2 h of movement per day [9–11]. Data from the GPS were imported into ArcGIS 10.2 software (ESRI, Inc.; Redlands, CA) and processed to create two measures of activity space: a standard deviational ellipse (SDE) and road network buffer (RNB) [12–15]. The SDE represents the area within which an individual spends 68% of their recorded time. A higher value indicates a larger proportion of time spent outside the home. We used a one standard deviation ellipse, which covers approximately 68% of all GPS-recorded points and is centered on the mean center of the point pattern. Its long axis is in the direction of maximum dispersion; its short axis is in the direction of minimum dispersion. Both one and two SDEs can be calculated for GPS data. The one SDE tends to capture the core or central location of the majority of the data better, while the two SDE captures the range a bit better. We did calculate the two SDEs as well, and our results were similar, likely because our sample is largely sedentary and spend much of their time at home. Therefore, we chose the one standard deviational ellipse as it most accurately depicts where the majority of time is spent. In contrast, the RNB represents the extent of space within which a person travels during the day. A larger value indicates a greater distance traveled away from the home. The RNB was calculated by buffering all GPS points at 50 m and dissolving these separate features into a single feature or space. We selected 50 m to capture the immediate vicinity around activity locations and travel routes. Both the SDE and the RNB yield a metric in square meters. Examples of GPS output are displayed in Fig. 1, panels a-c.

Accelerometer data were processed using the ActiGraph software. Movement was captured along the vertical axis in 15-s epochs, and non-wear time was defined as 60 min of consecutive zero counts. Participants were deemed to have a valid accelerometer record if they wore the monitor for 600 min per day for 4 or more days during the 7-day period. Several measures of physical activity were derived from these data including time spent sedentary, moderate-vigorous intensity movement (MVPA) and average number of steps accumulated per day (derived per the manufacturer’s default filter). Minutes of physical activity were estimated from accelerometer counts using cut points established by Freedson and colleagues (1998) for sedentary (<100 cpm), MVPA (>1952 cpm) and vigorous (≥5999 cpm) physical activity.

Other data processing and all statistical analyses were performed using SAS v9.4. Descriptive statistics were generated for all measures. We used Spearman correlation and linear regression to examine associations between outcomes. For multivariate models, to avoid issues with collinearity, we did not include any two candidate variables with a correlation coefficient > 0.6. We compared outcomes between subgroups by using Student’s t tests or Wilcoxon rank sum tests as appropriate. Descriptive plots of activity space and physical activity measures were created in R software. Data for activity space measures were log-transformed for plots.

## Results

**Fig. 2:**
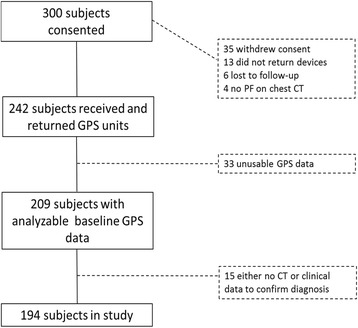
Flowchart of Enrolled Subjects

Of 300 subjects enrolled in the study, 242 received and returned devices, of whom 209 had valid, analyzable GPS data. Invalid GPS records were most commonly due to subjects not turning on or properly charging units. PF was not able to be confirmed definitively in 15 subjects who returned devices, and they were excluded. Thus, the study sample included 194 subjects (Fig. 2). Descriptive characteristics of the sample are displayed in Table 1. Over half the sample was male (56%), and the vast majority of subjects were Caucasian (95%). Nearly one third had IPF (30%) or a connective tissue disease related-PF (31%). Most subjects had comorbid conditions, and 43.8% of the sample (*N* = 85) had at least two comorbidities.

**Table 1:** Baseline Characteristics

**Characteristic**	**Result**	
**Sex**		
Male	108 (56)	
Female	86 (44)	
**Age**	65.8 ± 10.0	
**Race**		
White	184 (95)	
Black	5 (3)	
Asian	3 (1)	
Other	1 (1)	
**BMI**	28.3 ± 7.1	
**Ethnicity**		
Hispanic	11 (6)	
Non-Hispanic	183 (94)	
**Smoking Status**		
Current	3 (1)	
Past	102 (54)	
Never	85 (45)	
Missing	4	
**Employment**		
Job/Volunteer Outside Home	61 (33)	
Missing	9	
**Clinical Diagnosis**		
CTD	60 (31)	
cHP	16 (8)	
FPF	13 (7)	
IPF	59 (30)	
Other PF*	46 (24)	
**Had a Surgical Biopsy**		
Yes	74 (39)	
No	117 (61)	
Missing	3	
**Comorbid Conditions**		
CAD	42 (22)	
COPD	7 (4)	
DM	22 (11)	
GERD	99 (51)	
HTN	60 (31)	
OSA	36 (19)	
PE	7 (4)	
PH	14 (7)	

**Footnote:** cHP = chronic hypersensitivity pneumonitis; FPF = familial pulmonary fibrosis; IPF = idiopathic pulmonary fibrosis; CTD = connective tissue disease, including rheumatoid arthritis (*N* = 9), systemic sclerosis (*N* = 19), undifferentiated connective tissue disease (*N* = 22), antisynthetase syndrome/myositis (*N* = 10); CAD = coronary artery disease; COPD = chronic obstructive pulmonary disease; DM = type II diabetes mellitus; GERD = gastroesophageal reflux disease; HTN = systemic hypertension; OSA = obstructive sleep apnea; PE = pulmonary embolism; PH = pulmonary hypertension; *diagnosis in medical records “ILD”

Table 2 shows summary statistics for baseline values for the outcome measures. Spirometry performed within 6 months of enrollment suggested mild physiologic restriction (FVC 73.9 ± 15.4%). For the cohort as a whole, dyspnea was mild; fatigue scores were at the upper limits of normal; and cough-related QOL was relatively unimpaired.

**Table 2:** Summary Statistics for Physical Activity, Activity Space and Other Outcome Measures at Baseline

	*N*	*Mean* ± *SD*	*Median (IQR)*
**Accelerometer Measures**			
% Day Sedentary	193	66.2 ± 9.1%	65.5%(59.8-73.0)
% Day MVPA	193	9.4 ± 5.3%	9.3%(5.3-12.3)
Steps Per Day	193	7644 ± 2702	7497(5766-9261)
**GPS Measures**			
SDE (km2)	194	2319.4 ± 13,205.9	86.7(20.2-377.5)
RNB (km2)	194	44.7 ± 78.9	22.4(11.2-43.6)
Pulmonary Physiology*			
FVC%	154	73.9 ± 15.4	74.0(63.0-83.0)
DLCO%	142	62.9 ± 14.9	61.5(52.0-74.0)
**UCSD Questionnaire**	186	25.5 ± 19.5	22.0(9.0-36.0)
**FSS Questionnaire**	188	32.3 ± 16.1	31.3(18.0-45.5)
**LCQ Questionnaire**			
Physical	187	5.5 ± 1.1	5.8(4.9-6.5)
Psychological	187	5.7 ± 1.3	6.1(5.0-6.9)
Social	184	5.7 ± 1.4	6.3(5.0-6.9)
Total	184	17.0 ± 3.6	18.2(15.0-19.8)
**SF-36**			
PF	188	40.2 ± 10.2	40.4(32.0-48.8)
RP	188	41.2 ± 12.1	42.1(28.0-56.2)
BP	188	50.0 ± 9.8	50.8(41.8-55.9)
GH	188	40.1 ± 10.2	39.2(32.2-48.1)
SF	188	47.8 ± 10.5	51.7(40.9-57.1)
VT	188	47.4 ± 11.1	49.1(39.6-56.2)
RE	188	44.9 ± 12.9	55.3(34.3-55.3)
MH	187	51.3 ± 9.3	55.0(45.9-57.3)
Physical Composite	187	40.9 ± 9.5	42.0(33.3-47.7)
Mental Composite	187	50.7 ± 10.7	53.2(43.9-59.7)

**Footnote:** MVPA = moderate to vigorous physical activity; GPS = global positioning system; SDE = standard deviational ellipse; RNB = road network buffer; FVC% = percent predicted forced vital capacity; DLCO% = percent predicted diffusion capacity of the lung for carbon monoxide; UCSD = University of California San Diego Shortness of Breath Questionnaire; FSS = Fatigue Severity Survey; LCQ = Leicester Cough Questionnaire; SF-36 = Medical Outcomes Study Short-Form 36-item Questionnaire; PF = Physical Functioning; RP = Role Physical; BP = Bodily Pain; GH = General Health; SF = Social Functioning; VT = Vitality; RE = Role Emotional; MH = Mental Health:*performed within 180 days of enrollment

**Fig. 3:**
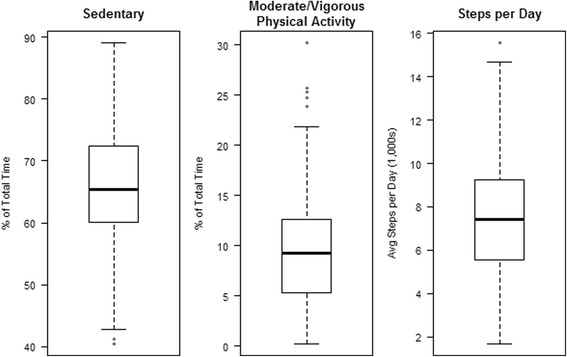
Distribution of Accelerometer-derived measures of physical activity. Footnote: horizontal line = median, box = interquartile range (IQR); whiskers = 1.5IQR; and dots = outliers

Subjects wore the accelerometers for GPS trackers for an average 5.7 ± 1.3 days and accelerometers for an average for an average 7.7 ± 2.6 days. Subjects spent a median 66.2% of total wake time (interquartile range [IQR] 59.8-73.0%) sedentary and 9.3% of wake time (range 5.3-12.3%) in MVPA (Fig. 3). Overall, subjects completed a median 7497 (IQR 5766-9261) steps per day. Wrist-worn accelerometers (*N* = 161) recorded significantly greater steps per day than waist-worn (*N* = 32) accelerometers (median 8238 ± 2513 steps/day vs 5357 ± 2367 steps/day, *p* = 0.0001). Wrist-worn accelerometers also recorded a significantly greater percentage of time in MVPA than waist-worn accelerometers (10.8 ± 4.6% vs 3.2 ± 3.0%, p = 0.0001). There was no difference in accelerometer data collected in winter versus non-winter months (winter: 7659.1 ± 2362.4 vs. non-winter: 7639.7 ± 2802.6, *p* = 0.97). According to the SDE, 15 subjects rarely ventured far, spending 68% of their time within two square kilometers of their homes. Another 7 subjects left home only three or four times during the measurement period and spent 68% of their time within five square kilometers of their homes.

There were statistically significant, predominantly weak correlations between physical activity and PROs (Additional file 1: Table S1). There were no statistically significant correlations between activity space and any other outcome measure (Additional file 1: Figure S1). Among subjects who were not employed (and did not volunteer outside the home), there was a trend toward statistical significance in correlations between GPS metrics and steps-per-day (r_s_(RNB,SPD) = 0.15, *p* = 0.08 and r_s_(SDE,SPD) = 0.17, *p* = 0.06). For the entire cohort, the correlation between cough and fatigue was moderately strong. The pattern of correlations was the same for the subgroup of subjects who wore the accelerometers on their waist (data not shown). On univariate analysis, there were several predictors of physical activity, assessed by steps per day (Table 3). Among several candidate models we developed, two are shown in Table 3. In the best model, which explained 34% of the variance in steps-per-day, fatigue was a strong, independent driver of physical activity, even controlling for age, body mass index, and diffusing capacity. There were no differences in outcomes between subjects with IPF and those with other forms of PF (Table 4).

**Table 3:** Linear regression showing candidate predictors of steps per day

	**Univariate Beta ± S.E.**	***P***	Multivariate Model 1** Beta ± S.E.	***P***	Multivariate Model 2** Beta ± S.E	***P***
**Intercept**			10,416 ± 1720	<0.0001	9285 ± 1269	<0.0001
**Male gender**	−674.0 ± 389.9	0.08				
**Age**	−37.4 ± 19.9	0.06	−47.8 ± 18.4	0.01	−46.0 ± 17.9	0.01
**BMI**	−56.7 ± 28.4	0.04	−32.8 ± 26.8	0.22		
**IPF diagnosis**	−16.5 ± 423.4	0.97				
FVC%*	5.1 ± 13.5	0.71				
DLCO%*	34.4 ± 14.4	0.01	20.4 ± 12.2	0.09		
**Number of comorbidities**	−391.8 ± 165.6	0.01				
**FSS**	−59.1 ± 11.3	<0.0001	−51.5 ± 11.7	<0.0001		
**LCQ**	160.7 ± 53.2	0.002				
**UCSD**	−33.3 ± 9.8	0.0008			−40.6 ± 9.1	<0.0001
**SF-36**						
**PF**	89.1 ± 17.9	<0.0001				
**RP**	64.5 ± 15.4	<0.0001				
**BP**	60.8 ± 19.3	0.001				
**GH**	73.8 ± 18.3	<0.0001				
**VT**	70.0 ± 16.8	<0.0001				
**SF**	65.7 ± 17.9	0.0003				
**RE**	45.9 ± 14.8	0.002				
**MH**	37.5 ± 20.8	0.07				
**Wrist accelerometer**	2881.4 ± 484.8	<0.0001	2974.6 ± 477.6	<0.0001	2993.8 ± 460.7	<0.0001
**Enrolled in winter**	19.5 ± 464.9	0.97				
**Lives in Colorado**	−298.2 ± 406.4	0.46				
**Employed outside home**	410.0 ± 423.5	0.33				
**Adjusted R-square**			0.34		0.27	

**Footnote:** *Within 120 days of enrollment; **Backward selection of candidate variables that included male gender, age, body mass index (BMI), percent predicted diffusion capacity of the lung for carbon monoxide (DLCO%), number of comorbid conditions, wrist vs. waist accelerometer wear, and fatigue (Model 1) or dyspnea (Model2); FVC% = percent predicted forced vital capacity; UCSD = University of California San Diego Shortness of Breath Questionnaire; FSS = Fatigue Severity Survey; LCQ = Leicester Cough Questionnaire; SF-36 = Medical Outcomes Study Short-Form 36-item Questionnaire; PF = Physical Functioning; RP = Role Physical; BP = Bodily Pain; GH = General Health; SF = Social Functioning; VT = Vitality; RE = Role Emotional; MH = Mental Health

**Table 4:** Comparison of P3F Outcome Measures for Subjects with IPF versus other clinical summary diagnoses

	**IPF (** ***N*** **= 59)**	**Non-IPF (** ***N*** = 134)*	***P***
**GPS Measures**			
SDE (km2)	106.0(25.5-712.2)	85.1(18.5-286.6)	0.25
RNB (km2)	30.0(10.9-60.9)	20.2(11.2-37.5)	0.09
**Accelerometer Measures**			
% Day Sedentary	67.1 ± 8.3%	65.8 ± 9.5	0.38
% Day in MVPA	8.5 ± 4.3%	9.7 ± 5.6%	0.15
Steps Per Day	7633 ± 2354	7649 ± 2851	0.97
**UCSD Questionnaire**	24.9 ± 16.0	25.8 ± 20.9	0.77
**FSS Questionnaire**	33.1 ± 15.1	31.9 ± 16.5	0.63
**LCQ Questionnaire**			
Physical	5.6 ± 0.9	5.5 ± 1.1	0.60
Psychological	5.7 ± 1.3	5.7 ± 1.4	0.87
Social	5.7 ± 1.3	5.7 ± 1.4	0.99
Total	17.0 ± 3.4	16.9 ± 3.8	0.87
SF-36**			
PF	40.0 ± 9.3	40.2 ± 10.7	0.87
RP	40.5 ± 11.6	41.6 ± 12.4	0.55
BP	52.6 ± 9.4	48.7 ± 9.8	0.01
GH	39.4 ± 9.6	40.5 ± 10.5	0.48
SF	49.4 ± 9.0	47.0 ± 11.1	0.14
VT	47.8 ± 9.9	47.2 ± 11.6	0.74
RE	45.2 ± 12.2	44.8 ± 13.2	0.86
ME	52.0 ± 9.2	51.0 ± 9.3	0.48
Physical Composite	41.0 ± 8.5	40.9 ± 10.0	0.94
Mental Composite	51.6 ± 9.7	50.2 ± 11.1	0.42

**Footnote:** Values = Mean ± SD or Median (interquartile range); *For one subject, questionnaire data were missing; **N = 129 for non-IPF; P3F = Participation Program for Pulmonary Fibrosis; MVPA = moderate to vigorous physical activity; GPS = global positioning system; SDE = standard deviational ellipse; RNB = road network buffer; FVC% = percent predicted forced vital capacity; DLCO% = percent predicted diffusion capacity of the lung for carbon monoxide; UCSD = University of California San Diego Shortness of Breath Questionnaire; FSS = Fatigue Severity Survey; LCQ = Leicester Cough Questionnaire; SF-36 = Medical Outcomes Study Short-Form 36-item Questionnaire; PF = Physical Functioning; RP = Role Physical; BP = Bodily Pain; GH = General Health; SF = Social Functioning; VT = Vitality; RE = Role Emotional; MH = Mental Health

## Discussion

In this study, we examined movement and other patient-centered outcomes in a cohort of patients with PF from known and unknown causes who had not been prescribed O_2_. The wearables gave a glimpse – and the GPS data, a first-ever look – into how subjects moved within their worlds and allowed us to examine how their movement related to other outcomes of known importance to PF patients. The accelerometer data revealed that day-to-day physical activity was surprisingly high in this cohort of PF patients. The GPS data confirmed great heterogeneity in activity space and showed that many subjects did not move far from home during the baseline measurement period.

Patients with PF may be profoundly limited in their day-to-day physical activity due to a variety of factors including dyspnea, fatigue, motivation or the physical constraints of O_2_. In this cohort with relatively mild PF (according to the mean FVC% and absence of the need for O_2_), although causation could not be established, as anticipated, better day-to-day physical activity (as determined by steps per day) was associated with less dyspnea, less fatigue and better quality of life scores across a variety of domains. The absence of significant differences in patient-reported outcomes between subjects with IPF and non-IPF diagnoses likely stems from the enrollment criterion that patients’ disease not be so severe as to require O_2_: subjects had relatively similar disease severity levels, regardless of diagnosis. Steps-per-day recorded by waist-worn accelerometers (5357 ± 2367 steps/day) were similar to results from subjects with GOLD stage II or III COPD (FEV1% = 40.0-64.8, steps/day = 5272 ± 1772 – 6600 ± 2331 steps/day) who wore ankle accelerometers in an observational, longitudinal study [16]. Startlingly, in a large, nationwide, US, 2-day study of waist-worn pedometers, among 261 subjects 60 years of age or older, the average number of steps per day was only 4027 (standard error 169) [17].

Recently, GPS trackers and other wearables have been used with increasing frequency to obtain a more granular view of patient movement [18, 19]; however, to our knowledge, GPS technology has not previously been used to study patients with chronic, parenchymal lung disease. We found no correlation between GPS data and any other outcomes, including accelerometer data. This suggests that patients who move outside the more immediate vicinity of their homes (greater SDE) are not necessarily more physically active than those who remain at – or closer to – home. In addition, activity space is likely related to many factors aside from disease severity, such as work outside the home, independence of living, and even ownership of a pet. Traditionally, investigators have examined physical activity and mobility with accelerometers. Our findings confirm that movement among PF patients is highly variable, that measures constructed from accelerometer and GPS data assess different aspects of movement: pedometers track physical, person-powered movement, whereas GPS will pick up “assisted” movement (e.g., traveling in a car, etc.). Regardless, the two wearables can be used in tandem to create a more complete picture of how patients move in the world.

Results for PROs and other outcome measures were as expected [1, 2, 20, 21]. Even among this cohort of patients with “mild” PF, fatigue – an often-overlooked symptom of PF – the was prominent, with 45% of the cohort reporting significant fatigue based on FSS scores greater than 35 points. Likewise, cough-related quality of life was significantly impaired in some subjects: 32 had LCQ total scores less than 13. SF-36 scores showed impairment in physical domains of health status, and dyspnea was moderately severe in nearly 15% of the cohort, based on the UCSD scale.

This study has limitations. Subjects were recruited from across the U.S., representing 34 different states, and were not required to visit the study site, thus precluding uniform and systematic collection of baseline clinical data. Although nearly a third of the cohort had IPF, the number of subjects with any one PF etiology was relatively small; this potentially limits the ability to generalize results to any specific subtype of PF. We lost approximately 13% of our sample due to invalid GPS data, which is comparable to other studies using GPS trackers. There are many potential reasons: because, by design, GPS units were mailed, subjects did not receive formal training in the operation of the units and had to rely on provided, written instructions. Even though both wearables were faceless (i.e., had no display), it is possible that wearing them induced subjects to be more active than they would have been otherwise. This may account for the higher-than-expected number of minutes per day in moderate to vigorous activity for our cohort as compared to a representative, general, U.S. adult population [22]. As anticipated [23], wrist-worn accelerometers recorded more steps per day than waist-worn accelerometers. This is due to accelerometers recording hand movements as steps. Although, correlations among outcomes were similar whether accelerometry data were generated from waist- or wrist-worn units, we recommend they be worn on the waist (or ankle, not the wrist) to give the most accurate step count and activity data. For GPS data, there is no way to derive wear time, because they turn off during long periods of sedentary time. Thus, we were forced to rely on patient diaries for GPS wear time. Diaries suggest that subjects wore them as we asked. An individual’s movement may be shaped by a variety of individual physical and non-physical constraints, the individual’s needs and preferences, the environment they live in (e.g., rural vs. urban settings, climate) as well as the resources available to them—all of which influence whether and how a person engages with the world around them. We attempted to assess and control for many of these factors, but there are some that we were unable to address. Finally, the study design and recruitment strategy likely selected motivated subjects comfortable with electronic gadgets and using a computer. We attempted to mitigate the influence of computer illiteracy by giving subjects the options to complete questionnaires on paper, which nearly a third of subjects did.

## Conclusion

Patients with PF face challenges in navigating the world; these challenges increase as their disease progresses. Disease-related symptoms, including dyspnea and fatigue, can impair movement but appear to be unrelated to activity space. Our study highlights the fact that GPS and accelerometry units capture different but important aspects of mobility. As wearable technologies are used with increasing frequency in clinical research, it will be important for investigators to understand the limitations of each modality. Future research on this cohort will include additional, longitudinal data. With this added wave of data, we hope that that GPS and accelerometry units help us to better understand whether and how O_2_ therapy affects movement and interaction with the world in patients with PF.

## Additional file

Additional file 1: Supplementary materials for: “Physical activity and activity space in patients with pulmonary fibrosis not prescribed supplemental oxygen.” The supplementary file contains one additional table of study data results: **Table S1.** Spearman correlation coefficients showing association between wearables and other outcomes. The file also contains one **Figure S1:** Relationship between Activity Space Measures (SDE and RNB) and Physical Activity Measures (% of time Sedentary and in MVPA). (PDF 186 kb)

## Abbreviations

FFS: Fatigue severity scale
GPS: Global positioning system
HRQL: Health related quality of life
IQR: Interquartile range
LCQ: Leicester cough questionnaire
MVPA: Moderate-vigorous intensity exercise
PF: Pulmonary fibrosis
QOL: Quality of life
RNB: Road network buffer
SDE: Standard deviational ellipse
SF-36: Medical outcomes study short form 36-item instrument
UCSD: University of California San Diego shortness of breath questionnaire
