# Supplementary material for: Physical activity and activity space in patients with pulmonary fibrosis not prescribed supplemental oxygen
Source: BMC Pulm Med. 2017 Nov 23;17:154. doi: 10.1186/s12890-017-0495-2 (PMC5701349; doi:10.1186/s12890-017-0495-2)
Supplement: Supplementary file 1 — Supplementary materials for: “Physical activity and activity space in patients with pulmonary fibrosis not prescribed supplemental oxygen.” The supplementary file contains one additional table of study data results: Table S1. Spearman correlation coefficients showing association between wearables and other outcomes. The file also contains one Figure S1: Relationship between Activity Space Measures (SDE and RNB) and Physical Activity Measures (% of time Sedentary and in MVPA). (PDF 186 kb) [file 12890_2017_495_MOESM1_ESM.pdf]

Supplementary materials

Physical activity and activity space in patients with pulmonary fibrosis not prescribed supplemental oxygen

Elisabeth Dowling Root, PhD[1,3]
Bridget Graney, MD[2,4]
Susan Baird[3]
Tara Charney, MPH[3,4]
Kaitlin Fier, MPH[3,4]
Marjorie Korn[4]
Mark McCormick[4]
David Sprunger, MD[4,5]
Thomas Vierzba[4]
Frederick S. Wamboldt, MD[4,5]
Jeffrey J. Swigris, DO, MS[3,4,5]

[1]Department of Geography and Division of Epidemiology, The Ohio State University, Columbus, OH, USA

[2]Division of Pulmonary Sciences and Critical Care Medicine, University of Colorado Anschutz Medical Campus, Aurora, CO USA

[3]Autoimmune Lung Center and Interstitial Lung Disease Program, National Jewish Health, Southside Building, Office #G011 1400 Jackson Street Denver, CO 80206, USA

[4]Participation Program for Pulmonary Fibrosis (P3F), Denver, CO, USA

[5]Division of Pulmonary, Critical Care and Sleep Medicine, Sleep & Behavioral Health Sciences Section, National Jewish Health, Denver, CO, USA

Funding: This project was funded via PCORI contract CE 12-11-4134 (Dr. Swigris PI). The views are those of the authors and not necessarily PCORI's.

To whom correspondence should be addressed:
Elisabeth Dowling Root
Department of Geography
The Ohio State University
1036 Derby Hall
154 North Oval Mall
Columbus, OH 43210 USA
Phone: 614-292-1882
Email: root.145@osu.edu

Table E1. Spearman correlation coefficients showing association between wearables and other outcomes.

Variable	SDE	RNB	Steps	ModVig	SedT	FVC%	DLCO%	FSS	LCQ	PF	RP	BP	GH	VT	SF	RE	MH	UCSD
**SDE**	1.000																	
**RNB**	0.915	1.000																
**Steps**	0.140	0.156	1.000															
**ModVig**	0.091	0.083	0.885	1.000														
**SedT**	-0.088	-0.092	-0.333	-0.283	1.000													
**FVC%**	0.033	0.065	0.064	-0.011	-0.145	1.000												
**DLCO%**	0.065	0.062	0.177	0.121	-0.072	0.394	1.000											
**FSS**	0.010	-0.034	-0.339	-0.267	0.145	-0.262	-0.196	1.000										
**LCQ**	-0.081	-0.027	0.223	0.143	0.045	0.257	0.191	-0.544	1.000									
**PF**	0.035	0.067	0.343	0.233	-0.062	0.250	0.289	-0.651	0.490	1.000								
**RP**	0.028	0.027	0.286	0.233	-0.050	0.197	0.243	-0.756	0.440	0.655	1.000							
**BP**	0.086	0.101	0.229	0.200	-0.124	0.073	-0.079	-0.488	0.305	0.360	0.379	1.000						
**GH**	-0.097	-0.043	0.271	0.215	0.040	0.153	0.201	-0.529	0.456	0.453	0.475	0.318	1.000					
**VT**	0.002	0.017	0.281	0.246	-0.083	0.254	0.137	-0.823	0.512	0.601	0.681	0.542	0.559	1.000				
**SF**	0.078	0.121	0.272	0.221	-0.141	0.114	0.157	-0.704	0.472	0.566	0.656	0.548	0.547	0.697	1.000			
**RE**	0.062	0.078	0.208	0.162	-0.119	0.145	0.125	-0.614	0.323	0.377	0.594	0.371	0.391	0.553	0.673	1.000		
**MH**	0.010	0.051	0.108	0.074	-0.090	0.136	-0.086	-0.494	0.345	0.320	0.342	0.340	0.410	0.548	0.586	0.454	1.000	
**UCSD**	0.008	-0.038	-0.249	-0.156	0.016	-0.280	-0.296	0.657	-0.548	-0.832	-0.648	-0.320	-0.491	-0.597	-0.548	-0.444	-0.378	1.000

Green=statistically significant accounting for multiple comparisons; SDE=standard deviational ellipse; RNB=road network buffer; Steps=average steps per day; ModVig=moderate to vigorous physical activity; SedT=average sedentary time; FVC%=percent predicted forced vital capacity; DLCO%=percent predicted diffusion capacity of the lung for carbon monoxide; FSS=Fatigue Severity Survey; LCQ=Leicester Cough Questionnaire; Medical Outcomes Study Short-Form 36-item Questionnaire domains (PF=Physical Functioning; RP=Role Physical; BP=Bodily Pain; GH=General Health; VT=Vitality; SF=Social Functioning; RE=Role Emotional; MH=Mental Health); UCSD=University of California San Diego Shortness of Breath Questionnaire

Figure E1: Relationship between Activity Space Measures (SDE and RNB) and Physical Activity Measures (% of time Sedentary and in MVPA)

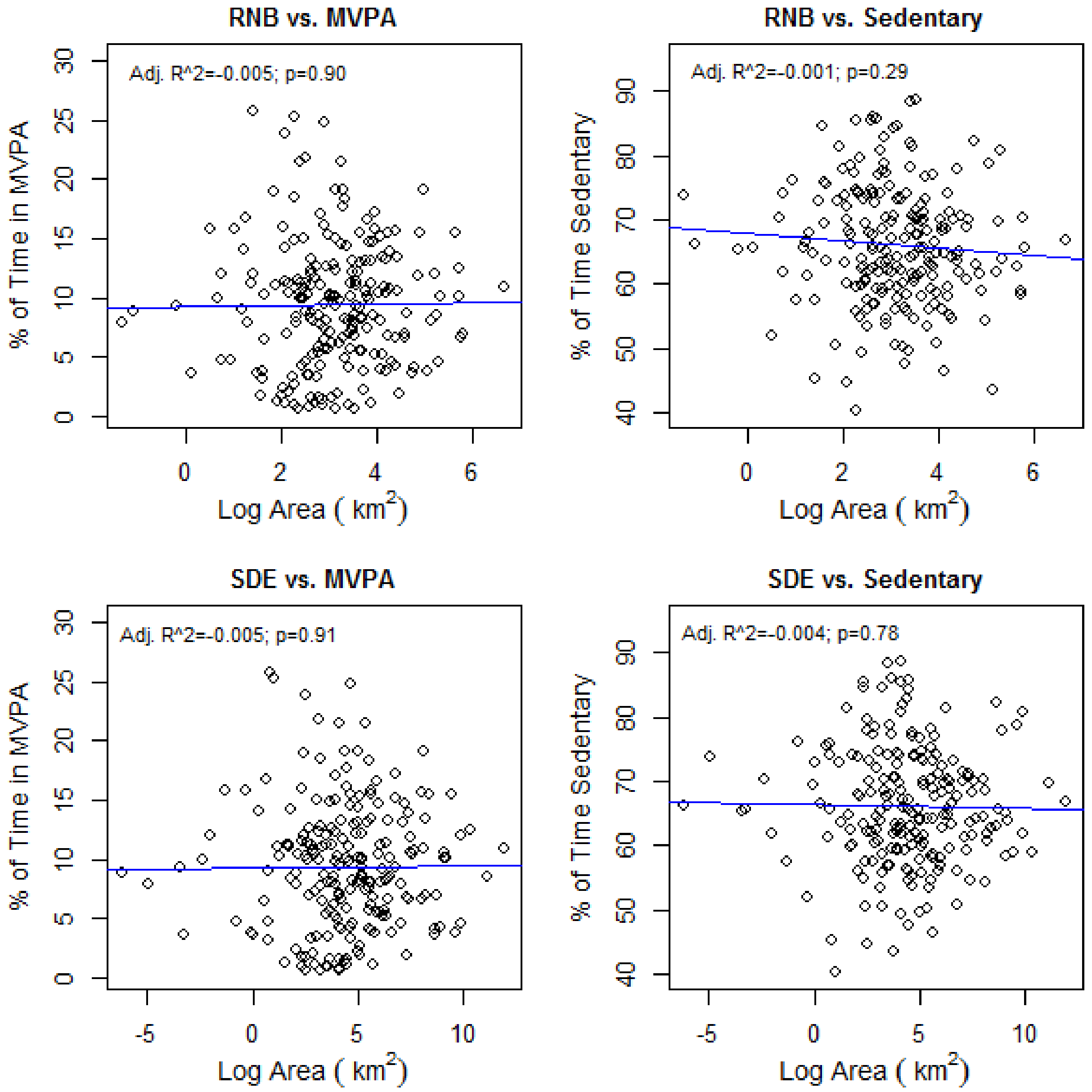

SDE=standard deviational ellipse; RNB=road network buffer; MVPA=moderate-vigorous physical activity
